# Boron: More Than an Essential Element for Land Plants?

**DOI:** 10.3389/fpls.2020.610307

**Published:** 2021-01-14

**Authors:** Greice Leal Pereira, João Antonio Siqueira, Willian Batista-Silva, Flávio Barcellos Cardoso, Adriano Nunes-Nesi, Wagner L. Araújo

**Affiliations:** Departamento de Biologia Vegetal, Universidade Federal de Viçosa, Viçosa, Brazil

**Keywords:** B deficiency, developmental programs, meristem regulations, metabolism, shoot growth, root growth

## Abstract

Although boron (B) is an element that has long been assumed to be an essential plant micronutrient, this assumption has been recently questioned. Cumulative evidence has demonstrated that the players associated with B uptake and translocation by plant roots include a sophisticated set of proteins used to cope with B levels in the soil solution. Here, we summarize compelling evidence supporting the essential role of B in mediating plant developmental programs. Overall, most plant species studied to date have exhibited specific B transporters with tight genetic coordination in response to B levels in the soil. These transporters can uptake B from the soil, which is a highly uncommon occurrence for toxic elements. Moreover, the current tools available to determine B levels cannot precisely determine B translocation dynamics. We posit that B plays a key role in plant metabolic activities. Its importance in the regulation of development of the root and shoot meristem is associated with plant developmental phase transitions, which are crucial processes in the completion of their life cycle. We provide further evidence that plants need to acquire sufficient amounts of B while protecting themselves from its toxic effects. Thus, the development of *in vitro* and *in vivo* approaches is required to accurately determine B levels, and subsequently, to define unambiguously the function of B in terrestrial plants.

## The Complexity That Lies Behind the Element Boron

Boron (B) was first described in the 1920s by a demonstration wherein *Vicia faba* L. (field bean) and other plants exhibited reduced root growth in the absence of B, but this could partially be rescued following the resupply of B (Warington, 1923). Later, it was suggested that B might play a pivotal role during the transition from aquatic to terrestrial environments, driving this evolutionary transition in plants (Lewis, 1980). Similarly, studies on the first vascular plant *Zosterophyllum shengfengense* suggested that B is primordial and originated in the root system in the terrestrial environment (Lewis, 1980).

In land plants, B plays important functions, including a structural role in cell walls, maintenance of plasma membrane functions, stimulation of reproductive tissues, and improvement of seed quality, and is influential in the biosynthesis of some metabolic compounds, such as antioxidants and polyphenols (Cakmak and Römheld, 1997; Marschner, 2012). Additionally, this element is involved in nucleic acid synthesis, phenolic metabolism, carbohydrate biosynthesis and translocation, pollen tube growth, and root elongation as well as it decrease indole-3-acetic acid (IAA) oxidase activity and, therefore, increased IAA content (Brown et al., 2002; Shireen et al., 2018; Landi et al., 2019). However, the molecular mechanisms underlying these functions remain largely unknown. Nevertheless, compelling evidence has demonstrated an elaborate system involved in both the uptake and transport of B in different plants (Miwa and Fujiwara, 2010). It was previously believed that the uptake process is exclusively passive and unregulated; however, it is now evident that plants detect the external and internal conditions of B, regulating and modulating the expression and/or accumulation of specific transporters in roots and shoots to maintain B homoeostasis in the plant (Miwa and Fujiwara, 2010).

**Figure 1:**
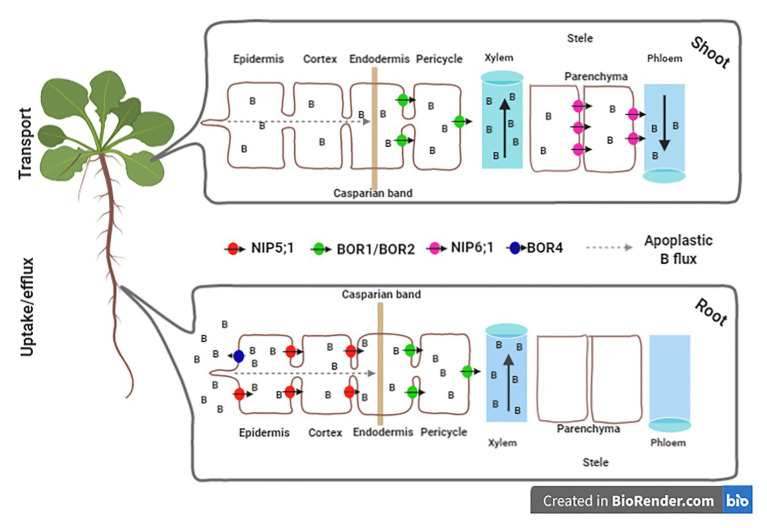
Overview of homeostasis of the boron (B) levels in plant cell mediated by transports. In *Arabidopsis thaliana* roots, B homeostasis is mainly based on three transport mechanisms across the plasma membrane. The first is the simple diffusion of boric acid (H_3_BO_3_), without charge through the lipid bilayers. The second mechanism represents the facilitated diffusion mediated by channels of the aquaporin family, such as NIP5;1. The third refers to the B movement by specific transports (e.g., BOR 1, BOR2, and BOR4). The uptake channels NIP5;1 is expressed in the plasma membranes of root cap and epidermal cells with outside/soil-facing polarity (Takano et al., 2006; Wang et al., 2017). NIP5;1 mediates the efficient radial transport of B from the soil through the epidermis and cortex to the endoderm. The Casparian band developed in the endoderm limits the apoplastic flow of B to the stele. BOR1 and BOR2 are expressed at the endodermis in meristematic and maturation zones (Miwa et al., 2013; Reid, 2014; Yoshinari et al., 2019). BOR1 and BOR2 are polarly localized in the stele-side plasma membrane domain (Takano et al., 2010; Miwa et al., 2013). BOR4 located in the root epidermis aid in the efflux of B from the root to the soil solution (Miwa et al., 2007). In shoots, NIP6;1 is necessary for the transport of B from xylem to phloem in nodal regions, being located in the parenchyma of the stele (Tanaka et al., 2016). Figures were created using the software biorender (app.biorender.com).

Recently, from another perspective, B was assumed to be a rather toxic element, causing substantial damage to plant cells even at low levels (Reid et al., 2004; Landi et al., 2019). Moreover, B essentiality is questionable considering that the responses to its deficiency are largely caused by the toxicity of phenylpropanoids (Lewis, 2019). It is noteworthy that alleles mediating tolerance to high levels of B in the soil remain in the wheat elite cultivar genomes following the selection of wild breeds cultivated by the first farmers in the Mediterranean region (Pallotta et al., 2014). Remarkably, these tolerance alleles are widespread in elite wheat cultivars developed in countries with soils containing extremely low levels of B. Evidence of a genetic distribution correlated to B levels in soils from different geographic regions suggests the possibility of alternative roles in contrasting environments. Collectively, this implies that combining B tolerance alleles with the level of B in the soil is important for mediating plant developmental programs. Accordingly, B can induce molecular pathways, which correspond to a series of actions among molecules in a cell that can turn genes on and off, thereby regulating developmental phase transitions (Figure 1). These transitions are involved in the coordination of the transition of plants to the adult phase (Redondo-Nieto et al., 2012; Lu et al., 2014; Xu et al., 2016; Kobayashi et al., 2018; Sakamoto et al., 2018).

Over the last century, several independent and complementary studies have provided compelling evidence for the involvement of B and its significance for plant development and growth. Remarkably, this aspect was recently contested based on metabolic responses that might be confused with B deficiency responses. Here, we summarize the complex B relationships in the soil, as well as how these relationships might influence an elaborate system of transporters governing B uptake from the rhizosphere. We further discuss the preferential B distribution and remobilization among tissues and their functional implications. Furthermore, we revisit the functions of B in mediating single genetic mechanisms in meristem cells, which might contribute to explaining the essentiality of this element. Finally, we provide compelling evidence supporting the essentiality of B in mediating plant developmental programs. We further posit that the development of new tools to precisely determine B levels *in vivo* is necessary to unequivocally demonstrate the essentiality of B.

## Boron Dynamics and Transport Inside Plants

The patterns of B distribution in the rhizosphere and B behavior in the soil directly affect its availability to plants. The soil solution contains predominantly boric acid (H_3_BO_3_) and borate anion [B(OH)_4_^−^], in which the chemical equilibrium strongly depends on the soil pH (Klochko et al., 2006). Another variable that strongly influences the physicochemical properties of H_3_BO_3_ is the ionization constant (pK_a_). Generally speaking, pK_a_ can be defined as the pH of a solution where the concentration of the undissociated species is equal to that of the ionized species, that is, [HA] = [A]. Thus, in solutions with pH lower than pK_a_, there is a higher H_3_BO_3_ concentration. At higher pH, the form B(OH)_4_^−^ predominates. Because of its small ionization constant (pK_a_ 9.25), H_3_BO_3_ is the predominant form under conditions of pH below the pK_a_ (Tanaka and Fujiwara, 2008).

Following the mass flow phenomenon, B is directed to the roots where this nutrient might be taken up from the soil solution (Marschner, 2012). Both metabolic and non-metabolic processes predominantly regulate the B uptake mechanisms. The mechanism of passive diffusion through the plasma membrane is considered an exclusive process for B uptake by roots (Nable et al., 1990; Brown and Hu, 1994; Hu and Brown, 1997). However, field results revealed significant differences in both the concentration and total B content between different species and genotypes cultivated under identical conditions (Nable, 1988). This suggests that the differences in B uptake between species are possibly caused by differences in membrane permeability to boric acid/borate (Huang and Graham, 1990; Hu and Brown, 1997) and that the absorption of B through lipid bilayers occurs through a combination of passive transport (diffusion) and possibly transport mediated by channels (Dordas et al., 2000). Intriguingly, by combining genetic and biochemical approaches, the presence of B-specific transporters was demonstrated not only in roots but also in leaves and reproductive organs (Takano et al., 2008; Chatzissavvidis and Therios, 2011).

The activity of B transporters is tightly regulated in response to the levels of B in the soil solution, optimizing B uptake and use, and maintaining nutrient homoeostasis in different plant tissues (Takano et al., 2008; Yoshinari and Takano, 2017). Briefly, plants control the capture and transport of B through the rapid regulation of two different classes of B transporters, namely, the channels of the MAJOR INTRINSIC PROTEIN (MIP) family and the boric acid/borate transporters of the BOR family (Matthes et al., 2020). The Nodulin 26-like intrinsic protein (NIP) family contains the major B transporters, including the root transporters NIP5;1 (boric acid channel) and BOR1 (boric acid/borate exporter), which are assumed to be the proteins responsible for maintaining B homoeostasis (Takano et al., 2008). NIP5;1 is located preferentially on the plasma membrane, with its polarity facing the soil side (Takano et al., 2006; Wang et al., 2017), whereas BOR1 is also located on the plasma membrane but with its polarity toward to stela, playing a key role in xylem loading (Wakuta et al., 2016; Yoshinari et al., 2019). According to the activities of these transporters, the directional and radial B transport from the soil solution into xylem is mediated under low soil levels of B (Sakamoto et al., 2011). From there, B is mobilized into the xylem and transported through the flow to the shoot (Matthes et al., 2020).

In contrast, under excess B, the transporter BOR4 is involved with B exclusion from cells and tissues, enhancing tolerance to B toxicity (Miwa et al., 2007; Yoshinari et al., 2019). Additionally, NIP6;1 corresponds to a channel that facilitates the permeability of boric acid across the plasma membrane and was recently considered completely impermeable to water (Tanaka et al., 2016). NIP6;1 transcript accumulation occurs in response to B deficiency. This transporter is predominantly expressed in the stem node regions, mainly in the phloem region (Tanaka et al., 2016). Thus, a sophisticated system for B uptake and translocation based on the families NIP and BOR is present in land plants, in which the expression of NIP5 isoforms is highly induced in both roots and shoots (Diehn et al., 2019; Figure 1).

In agreement with the differential activity of B transporters across distinct plant organs, it has been demonstrated that, with the aid of mathematical and computer science tools, the function of B transporters can be effectively predicted and experimentally validated in different species. For instance, by using the FORESTS database, a BOR1 homolog was found in *Eucalyptus* (Domingues et al., 2005), whereas the recent usage of 18 different plant genomes allowed the identification of 80 and 34 homologs of BOR1 and NIP5;1, respectively (Ozyigit et al., 2020). In addition, Kurt and Aydın (2020) identified and characterized five BOR1 transporters in potato (*Solanum tuberosum*) by using an *in silico* study. By performing an extensive co-expression network of each transporter, it was revealed that (i) there is a potential interaction between B transporters and genes involved in cell wall and (ii) a co-expression between StBOR1 transporters and plant immunity system (Kurt and Aydın, 2020). Thus, the identification of similar sequences for B transporters in different plants species reinforces the motion that those genes are most likely members of highly conserved gene families in plants that are expressed in different plant tissues/organs and involved in many biological processes (Yoshinari and Takano, 2017; Diehn et al., 2019). We posit that the combination of experimental research with theoretical and predictory approaches are fundamental to adequately understand the precise regulation and function of B transporters in both model and non-model plant species.

Using a mathematical modeling approach, it has been recently demonstrated that the precise regulation of the abundance of transporters ensures constant B concentration in the root cells (Matthes et al., 2020). Accordingly, transcripts encoding NIP members were also detected in floral tissues and showed expression patterns dependent on the developmental stage, which appeared to contribute to B distribution in the flowers of B-deficient plants (Diehn et al., 2019). In maize (*Zea mays*), double mutants for functional homologs of BOR1 (*RTE* and *RTE2*) were characterized by a slow developmental time of the shoot apical meristem (SAM), which displays reduced fertility and small ears (Chatterjee et al., 2014, 2017). It appears reasonable to assume that an elaborate genetic network allows land plants to cope with B levels *via* a tightly regulated response to this nutrient.

Since large amounts of gene expression data are currently available, it seems reasonable to anticipate that the growing power of bioinformatics coupled with computational resources will open new avenues to discover proteins involved in B transport. In fact, the combination of computational and imaging tools is becoming a recurrent feature in studies of complex and dynamic characteristics in plants (Tsaftaris et al., 2016). However, the fundamental challenge is to understand the mechanisms underlying changes in time and space of B transporters. By coupling mathematics and experimental approaches, a two-dimensional cross-sectional model of the *Arabidopsis thaliana* root meristem was developed taking into account spatial nuances of the location, polarity, and intensity of the B transporters (Shimotohno et al., 2015). However, the levels and activity of the transporter were static. Nonetheless, a rapid regulation of B transporters, through the dynamic regulation of these transporters, was further observed (Sotta et al., 2017). Furthermore, a mathematical model was generated by capturing the spatio-temporal distribution of B through root cross sections (Sotta et al., 2017). In summary, the mapping of the vast majority of B transporters was found along the roots although B transport can occur at different rates along the roots since certain transporters have distinct locations.

Significant progress has been recently obtained with the use of X-ray crystallography allowing the establishment of the structure-function relationship of proteins involved in the transport of B, that plays an important role in the perception of B levels in different tissues (Hrmova et al., 2020). Understanding the three-dimensional (3-D) structure of a transporter provides crucial insights into its function, identifying the main motifs or residues that may interact with potential substrates. Notwithstanding this fact, the results of this model are rather incomplete and thus the combination of multidisciplinary studies to fully elucidate the precise molecular mechanisms, structural dynamics, and regulation of B transporters is seemingly still required (Hrmova et al., 2020). Under a high supply of B, the BOR1 accumulated in the plasma membrane is rapidly ubiquitinated and transported to multivesicular bodies (endocytosis), and subsequently targeted for the vacuole for degradation (Takano et al., 2010; Wakuta et al., 2016; Yoshinari et al., 2018). Recently, Yoshinari et al. (2019) observed that AP2-dependent endocytosis maintains the polar localisation of BOR1 to support plant growth under low-B conditions, whereas the B-induced vacuolar sorting of BOR1 is mediated through an AP2-independent endocytic pathway. This response was assumed to be important for plant acclimatization to high B conditions (Tanaka and Fujiwara, 2008). Curiously, B-transporter genes specific to wheat roots (*Triticum turgidum* L. var. Durum) which modulate adaptation for B in the soil, exhibited allele origin and dispersion worldwide, which is important for the tolerance to distinct B levels (Pallotta et al., 2014).

Thus, these tolerance alleles display a large natural variability mediating B responses, which appears to have been remodeled following selective breeding of elite cultivars because of the contrasting environments during selection (Pallotta et al., 2014). Collectively, compelling evidence obtained during the last decades indicates intrinsic relationships between B transporters and plant development in general, which exhibit evolutionary patterns that drive plant breeding, highlighting the importance of this element, and consequently, in contrast with characteristics of a toxic element. Remarkably, there is virtually no current evidence of any plant that evolved specific morphoanatomical or genetic mechanisms to deal with toxic levels of B, rather there is only the regulation of B transporters.

## Boron Translocation and Distribution

Following B uptake by the roots, transpiration drives B translocation through xylem cells (Marschner, 2012). By analyzing B tolerance, beyond intrinsic regulation mediated by transporters in distinctly tolerant species, it was possible to verify that differential B tolerance is also attributed to the ability to restrict nutrient translocation from roots into shoots, allowing a high B accumulation in roots. Plants with this capacity have been indicated for phytoremediation in areas with high levels of B (Xin and Huang, 2017).

Boron can also be transported *via* the phloem, allowing its translocation between vegetative and reproductive tissues (Camacho-Cristóbal et al., 2008), although this is highly variable among species. The mobility is due in part to B complexation with polyols. Accordingly, in restricted mobility plants, B is relatively immobile in phloem; therefore, the most common deficiency symptom can be observed in young leaves and meristematic tissue, which is related to the death of the apical meristem (Will et al., 2011; Marschner, 2012). Additionally, in certain plant species, including apples, nectarines, *Arabidopsis*, and citruses, B can be transported in the phloem in quantities that are sufficient to meet plant requirements (Brown and Hu, 1996; Takano et al., 2001; Wu et al., 2019). The transport of B *via* phloem occurs by the formation of B-diol complexes, which are important for nutrient remobilization among tissues (Brown and Hu, 1996; Hu et al., 1997). B can easily bind to cis-hydroxyl groups of sugar alcohols (mannitol and sorbitol), allowing B to be transported through the phloem (Reid, 2014), yet the translocated amount depends on the plant B status and the synthesis of photoassimilates (Du et al., 2020). It seems reasonable to suggest that the molecular aspects involved in both B translocation and remobilization are likely a critical knowledge barrier to B nutrition that must be further investigated to fully elucidate the essential nature of B for land plants.

The precise determination of B at the cellular level has not been achieved, given that the actual B measurements do not consider the dynamics of the element and the differential affinity of B for each B transporter. It has been suggested that the cytological content of B is the only B detection site, but detailed studies on B variation in other cell sites are lacking, such as in organelles. Therefore, it is clear that more detailed and high-resolution techniques are required and must consider the dynamics of this element in both space/time. Furthermore, its dynamics in living cells should be investigated. We posit that this may be achieved by the development of genetically encoded sensors or chemical sensors for H_3_BO_3_, similar to nanobiotechnology, which allows the development of intelligent plant sensors that communicate chemical signals and the physiological and nutritional state of plants (Giraldo et al., 2019). To this end, the conventional approach suggested is the measurement of B stable isotopes, namely ^10^B and ^11^B, allowing the analysis of B pools in plant tissues (Dannel et al., 2000). The recent application of ablation laser-ICP-MS (inductively coupled plasma mass spectrometry) allowed the development of a mathematical model that enabled the analysis of B distribution along roots of *A. thaliana* at an extremely high resolution (Shimotohno et al., 2015). To overcome the challenges of B detection in different plant tissues, the fluorinated-18 4-fluorophenylboronic acid radiotracer approach ([18F] FPBA) was recently used (Housh et al., 2020). The images obtained by autoradiography provided insightful information on the patterns of static B location in both root and shoot tissues (Housh et al., 2020), revealing different locations of B distribution. Briefly, both the root tip and the stretching zone are the ones that exhibited the most intense sign of the tracer with a region between the two devoid of any sign, showing a clear demand for B in these regions (Housh et al., 2020). Consequently, future studies using such tools are important to determine element distribution throughout the plant and behavior of specific tissue levels.

The revolution recently obtained by the development and characterization of genetically encoded biosensors for cytosolic H_3_BO_3_, based on the fluorescent proteins of the transporters NIP5; 1 and BOR1 (Fukuda et al., 2018), has contributed to the first visualization of B in living plant cells. The fluorescence intensity in roots and shoots of the transgenic plants was high under B-limiting conditions and gradually decreased with an increasing concentration of B (Fukuda et al., 2018), which confirms sensor functionality. Such biosensors allow not only the visualization of the distribution but also the dynamics of B at physiologically relevant levels in various tissue and cell types. As a result, these sensors can be used by a wider range of purposes, including determination of whether the phenotype studied is influenced by differences in the concentration of B, demonstrating the importance of B for plant growth and development. Therefore, it is clear that the revolution afforded by next-generation sequencing means that appropriate tools and resources are becoming available to fully unravel the function of B in different crops. Thus, it becomes necessary that tools such as *in situ* hybridizations (Thisse and Thisse, 2008), promoter-gene fusions (Van Dyk et al., 2001), metabolite sensors (Wang and Lei, 2018), single-cell sequencing (Hwang et al., 2018), and laser microdissection (Nelson et al., 2006) be collectively considered. We anticipate that this will open new avenues for understanding B dynamics and will ultimately allow increased plant yield by tailoring approaches to tap into more specific attributes of desired species.

## Boron Mediating Developmental Transitions in Both Shoots and Roots

**Figure 2:**
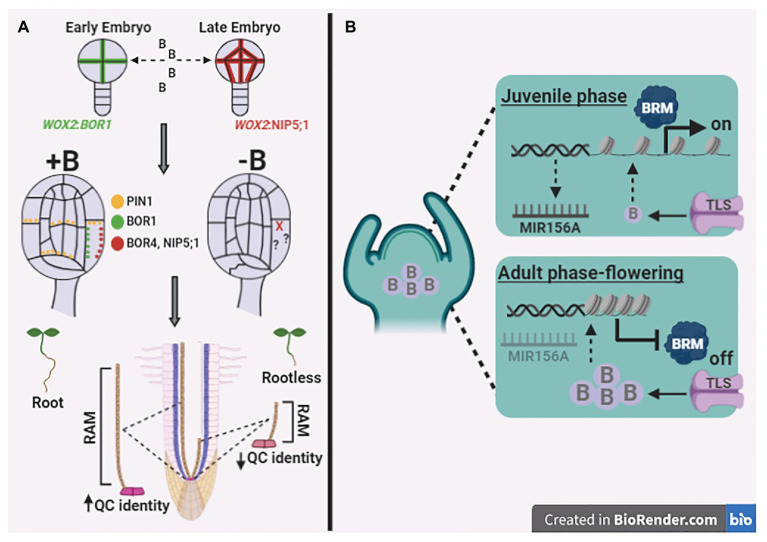
Boron mediating crucial developmental processes in roots and shoots. **(A)** The asymmetric cell divisions at early embryos exhibit a remarkable pattern related to BOR1, whereas NIP5;1 are the better marker for these divisions on late embryogenesis, suggesting the B role in orientating embryo development. Curiously, the B deficiency induction on mature embryos alters not only BOR1 and NIP5;1 gene expression patterns but also disrupts auxin transport among the different cell-types impairing root apical meristem (RAM) formation, which result in a rootless *A. thaliana* plants. Furthermore, B deficiency on mature plants reduce length and identity of quiescent center (QC) at RAM, which altogether suggest that B display influence on overall root developmental stages. **(B)** Shoot apical meristem (SAM) exhibits the B transport differently regulated according with developmental phase, the TRUNCATED LEAF SYNDROME (TLS) transporter remobilize low B levels into SAM at juvenile phase. Apparently, the reduced levels of B enables the activity of Brahma (BRM) chromatin remodeler allowing the expression of miR156A, which arrest the flowering transition in juvenile tissues. The adult phase transition to flowering would be mediated by the increase on B levels at SAM, which would promote blocking of BRM activity, which represses miR156A expression releasing flowering induction. Dotted arrows indicate mechanisms not completely elucidated. Figures were created using the software biorender (app.biorender.com).

The indirect concept of nutrient essentiality posits that, in the absence of determined plant nutrients, plants do not complete their life cycle (Arnon and Stout, 1939). In this context, developmental transitions are required for land plants to complete their life cycle, and these transitions are regulated through the proliferation and differentiation of cells. Recently, the organogenesis of both zebrafish (*Danio rerio*) and *A. thaliana* are B dependent, as revealed by alterations in N-glycosylation patterns that arrest cell fates during the development of both species (Reguera et al., 2019). Furthermore, by mimicking B depletion using phenylboronic acid (PBA), it was observed that *A. thaliana* wild type plants treated with PBA were similar to the rootless mutant *monopteros*, wherein PBA disrupts auxin transport dynamics during early embryo development (Matthes and Torres-Ruiz, 2016). Briefly, B depletion culminated with the development of irregular cotyledons, dramatic deformations of the vascular system, large vacuoles, and a reduced number of epidermal cells with the complete absence of root apical meristem (RAM; Matthes and Torres-Ruiz, 2016). Similarly, B transporters (e.g., BOR1 and NIP5;1) were used as reliable markers to study cell polarity axes during embryogenesis, whereas other genes failed to reveal the precise localisation of cell membranes resulting from asymmetric cell divisions in *Arabidopsis* embryos (Liao and Weijers, 2018). Indeed, BOR1 is an optimal gene marker for early embryo cells, whereas NIP5;1 provides more stable signals in late embryonic cells (Liao and Weijers, 2018; Figure 2A). Collectively, these results suggest an important role for B in mediating embryo development owing to B transporter localisation, which further indicates asymmetric cell division. Notably, highly specific induction of B transporters has been described for RAM and shoot apical meristem (SAM; Durbak et al., 2014; Sakamoto et al., 2019). Thus, it seems reasonable to suggest that B is most likely a direct regulator of cell division and differentiation, particularly at these specific sites, which is consistent with the fact that B is an essential micronutrient.

The preferential distribution of B in developing tissues of rice (*Oryza sativa*) is seemingly regulated in consonance with the expression of *NIP*, *OsNIP3;1* (Shao et al., 2018). Accordingly, knockout plants for this gene exhibited reductions in B content in newly developed leaves coupled with improved B translocation to old leaves, suggesting that OsNIP3;1 plays a key role in the distribution of B in developing tissues (Shao et al., 2018). Furthermore, it has been demonstrated that B contributes to the maintenance of the identity of the root quiescent center (QC) and that B deficiency dramatically inhibits cell proliferation (Poza-Viejo et al., 2018). Cell division was strongly affected by B deficiency in the DNA replication phases in the RAM (Poza-Viejo et al., 2018). Furthermore, B deficiency reduced not only brassinolide accumulation but also RAM size and length of mature cells. Remarkably, these responses are dependent on the downregulation of brassinosteroid (BR) signaling genes, regardless of B transport and translocation (Zhang et al., 2019). Exogenous application of 24-epibrassinolide (eBL), a bioactive BR, rescued root growth inhibition under B deficiency, and application of the BR biosynthesis inhibitor, BRZ, aggravates root growth inhibition of wild type plants under B deficiency. Taken together, these results indicate that B deficiency downregulates BR signaling to inhibit root growth. B starvation positively regulates the expression of SHORT ROOT (SHR) and SCARECROW (SCR) genes, which can further induce the expression of miR 165 enabling *PHABULOSA* (PHB) to reduce the influence of cytokinins on root development (Lu et al., 2014). Under low B levels, NIP5;1 facilitates B influx into the roots, whereas protein activity is increased in the RAM following the transference of roots from low B levels to optimal B levels (Gómez-Soto et al., 2019). Together, these studies demonstrated that both meristem activity and fate are regulated in response to B levels, and collectively suggest that this micronutrient is of pivotal significance in mediating the developmental phase transitions of plants.

Mechanisms associated with root organogenesis are extremely sensitive to B deficiency, and they are likely regulated through B signaling (Abreu et al., 2014). By using mathematical modeling and further experimental validation, it was established that B flux does not display a continuous increase from root tips toward the mature zone (Shimotohno et al., 2015). Accordingly, B absorbed at the root tip is likely used only at this root site, whereas the mature root zones display higher importance to drive B transport for the shoot (Shimotohno et al., 2015). It is also important to mention that B acts by blocking the accumulation of aluminium (Al) in the apical root zone (Li et al., 2018), where Al might induce QC differentiation and reduce cell division and root elongation. This is in agreement with the hypothesis that B plays a protective role in the RAM. Moreover, the reduced cell differentiation observed in *Medicago sativa* root nodules under B deprivation indicated that B plays specific functions in the initial phases of root organogenesis (Reguera et al., 2009). Additionally, B increases the degree of methyl esterification of pectin in the root apex, indicating higher plant resistance to Al toxicity because of increased cell wall plasticity (Riaz et al., 2018).

Genomic factors that induce cell differentiation and are involved in damage to DNA and DNA double-strand breaks (DSB) are triggered by high levels of B in plants (Hu et al., 2016). DSB induced under toxic B levels might be mitigated through the degradation of BRAHMA (BRM) protein, given that BRM binds to acetylated histone residues opening chromatin and causes to be DNA more exposed to B-related damage (Sakamoto et al., 2018). Additionally, BRM temporally regulates the expression of miR156, the master regulator of the transition from the juvenile to the adult phase, and as such, mutations in BRM accelerated the exit from the vegetative phase (Xu et al., 2016). B transport is critical for vegetative and reproductive maize development, where Tassel-Less1 (TLS1) protein facilitates meristematic B transport, which appears to be fundamental to meristem fate and inflorescence development (Durbak et al., 2014). Briefly, during the juvenile phase, there is an increased activity of BRM, allowing the expression of miR156A, which inhibits TLS and interrupts the flowering transition. On the other hand, the transition from the adult vegetative phase to flowering appears to be regulated by B transport, which reduces BRM activity and miR156A expression by releasing the flowering-TLS induction (Figure 2B).

Flowers are singular organs representing the last developmental phases; therefore, it is not surprising that efforts should be made to understand flower development and the roles B plays in this process, which is an exciting yet understudied field of plant biology. It has been demonstrated that NIP7;1, a facilitator of boric acid transport, is predominantly expressed in young flower anthers in a narrow developmental window (Routray et al., 2018). Accordingly, *nip7;1* mutants exhibit several defects in reproductive structures (Routray et al., 2018), indicating that proper control of B homoeostasis in both meristem and reproductive organs is critical for the biological success of vascular plants. Thus, this evidence suggests that obtaining and maintaining an optimal B level in flower-related tissues is often imperative for successful plant reproduction.

Because B has been described as an essential micronutrient (Warington, 1923), substantial advances in our understanding of B-related pathways have been achieved illustrating the role of B in plants. Nevertheless, Lewis (2019) argued against B essentiality for land plants by proposing an alternative point of view over the direct metabolic effect of B. Briefly, Lewis (2019) suggested that B is, and always has been, potentially toxic for plants and, perhaps more importantly, that this attribute must be fully avoided for normal growth, development, and reproduction. This assumption relies on the fact that not only B but also phenolics (compounds considered toxic for cellular metabolism) are interconnected. Thus, plants must have evolved the ability to mitigate adverse effects of both B and phenolics by chemical (such as organic complexes: *cis*-diols for B and lignins for phenolics) and physical (movement into vacuoles/apoplasts) sequestration (Lewis, 2019). Hence, B complexes formed in the cell wall are most likely a mechanism allowing detoxification of such compounds and cannot be presented as evidence of B essentiality. Here, we add further complications to this assumption given that our current knowledge concerning the micronutrient gradient and distribution among distinct organs and tissues is rather limited and requires significant advances in nutritional microscopic techniques (e.g., development of micronutrient specific sensors and nutritional living-microscopy). We posit here that the temporal control of micronutrient levels in different developmental phases could help to unequivocally describe the novel and specific roles of B. It is reasonable to assume that even minimal changes in the levels of B in specific organs (e.g., meristem, anthers, and flowers) have probably remained unnoticed in several studies; thus, this has led to the proposition that B is not an essential micronutrient. Although Lewis (2019) suggested that B is a toxic element with which plants have evolved to cope, we believe that the metabolic reprogramming suggested still must be unequivocally confirmed. One possible way is to use mutant plants in the biosynthetic pathways of the free neutralizing agents (e.g., polyphenols) suggested and investigate their response to different B levels, in conjunction with precise quantification methods. In this case, one must expect that such plants will display toxic effects at much higher B concentrations, without changing the levels of these compounds.

In contrast to the premise of Lewis (2020), we strongly argue that the gradually evolving function of B in the regulation of meristem fate discussed above highlights the potential role of this micronutrient at very low doses and during a very short development window. Therefore, we postulate that B essentiality should not be discussed only in the context of both the biomolecule constitution and from an unnoticed metabolic viewpoint, but also regarding its importance for specific cell types in RAM and SAM (Figure 2). It is reasonable to assume that B is likely able to induce cell proliferation and differentiation, triggering the proper development of vascular plants. Although our theoretical growth proposal is rather distinct from the theoretical metabolic mechanism postulated by Lewis (2020), we believe that this inference should be seriously considered when designing strategies to test B essentiality.

## Conclusion and Future Perspectives

In recent years, the importance of B in plant growth and development has attracted much attention because its specific and complementary functions are not fully understood. However, significant advances have attested to the essentiality of this element for land plants. Nevertheless, Lewis (2019) purposely not only challenged the essentiality of B in the conventional sense but also speculated that it is toxic and as such cannot have a primary role. Although the biological speculation of such a statement remained elusive, several exciting and testable research avenues were recently provided by Lewis (2019, 2020) and Matthes et al. (2020).

We further provide another alternative to unequivocally demonstrate the essentiality of B. Once B is absorbed by the roots, it is preferably distributed to developing tissues, such as meristems and reproductive organs. Although we cannot rule out the metabolic mechanism suggested previously (Lewis, 2019), this differential B distribution provides at least circumstantial evidence highlighting the potential role of B in mediating plant development programs, by promoting the transition from the vegetative to reproductive phase (Figure 1), as well as enabling land plants to complete their life cycle. To accurately understand the role of B and thus convincingly prove its essentiality, we argue the importance of developing new diagnostic tools that allow the detection of minimal changes in B levels in different tissues and specific cells. The discussion highlighted both in Lewis (2019) and here should attract research from different but complementary fields to investigate hypotheses and add analytical techniques to the “parts list” of B functions to test their application and relevance. Although the application of these techniques requires substantial financial investment, it is highly possible to bring returns in the form of improved mechanistic functions of B.

Neither Lewis (2019) nor we have provided experimental evidence to unequivocally demonstrate B essentiality (or not). However, it opens several research avenues that should be pursued. Lewis (2019) expertly presented a theoretical proposal for the metabolic mechanism by which B toxicity is not only overcome but also put to reasonably good purpose in particular circumstances. Future directions of research are also accurately presented with several independent hypotheses. We further add complexity to this exciting discussion here by focussing on the role played by B in the regulation and control of axillary meristem fate. Future research is required to fully elucidate the role of B in the cell division of the quiescent center, and several open questions remain: What is the crosstalk and how can it target endoreplication? Does this response change with different photoperiods? How are the B transporters regulated during the day? Which oscillator modulates B and mediates growth responses? By answering these questions, we will likely close gaps in our current understanding of how B works within land plants. Understanding the mechanisms behind the accepted (and challenged) functions of B may help to elucidate how and to what extent B is important, and ultimately contribute to an enhanced understanding of its biological function, with significant practical applications in agriculture.

## Author Contributions

GP and WA organized and wrote the manuscript. JS, WB-S, FC, and AN-N provided critical evaluation and edited the text. All authors contributed to the article and approved the submitted version.

### Conflict of Interest

FC is an agronomist at U.S. Borax | Rio Tinto, a borate supplier company. U.S. Borax | Rio Tinto had no role in study design, data collection and analysis, decision to publish, or preparation of the manuscript described here.

The remaining authors declare that the research was conducted in the absence of any commercial or financial relationships that could be construed as a potential conflict of interest.
